# *PLOS Biology* 2018 Reviewer and Editorial Board Thank You

**DOI:** 10.1371/journal.pbio.3000177

**Published:** 2019-02-27

PLOS and the *PLOS Biology* team want to sincerely thank all of our Editorial Board Members, Guest Editors, and Reviewers for the journal in 2018. Your contributions of time and expertise support your research community, advance scientific progress, and continue to make *PLOS Biology* a leader in its field. This past year, *PLOS Biology* received the assistance of 173 Editorial Board members, 188 Guest Editors, and 2,057 Reviewers, who handled 2,009 manuscripts that resulted in 407 publications.

We’re deeply grateful to all of our volunteers whose dedicated efforts support *PLOS Biology* and Open Science. Thank you all for your work!
